# 

**Published:** 1888-06

**Authors:** 


JUNE, 1888.

### CONTENTS.

ORIGINAL PAPERS:— Page

Intubation of the Larynx. By JOHN DACRE, M.R.C.S. ... ... ... ... ... 73

Notes on Disinfection. By D. S. DAVIES, M.B. Lond., D.P.H. Cantab. ... ... ... ... ... ... ... ... ... ... ... ... ... ... ... 83

Menorrhagia, etc., and Hygienics. By JOHN MEREDITH, M.D. Edin. ... 102

CLINICAL RECORDS:—

A Case of Suicidal Perforation of the Stomach by a Red-hot Iron, with Remarks. By LOCKHART STEPHENS, M.R.C.S. ... ... ... ... ... 113

Notes of Out-Patient Cases. By J. MICHELL CLARKE, M.A., M.B. Camb., M.R.C.P. Lond. ... ... ... ... ... ... ... ... ... ... ... ... 120

REVIEWS OF BOOKS ... ... ... ... ... ... ... ... ... ... ... ... ... ... ... ... ... 134

NOTES ON PREPARATIONS FOR THE SICK ... ... ... ... ... ... ... ... ... 140

PERISCOPE ... ... ... ... ... ... ... ... ... ... ... ... ... ... ... ... ... ... 140

SCRAPS ... ... ... ... ... ... ... ... ... ... ... ... ... ... ... ... ... ... ... 151

